# Real-World Outcomes and Prognostic Factors in Patients with Radioiodine-Refractory Differentiated Thyroid Cancer Treated with Sorafenib: A Multicenter Study

**DOI:** 10.3390/jcm15134880

**Published:** 2026-06-23

**Authors:** Suheda Atas Ipek, Sendag Yaslikaya, Ismail Oguz Kara, Tolga Koseci, Ertugrul Bayram, Esra Asarkaya, Hatice Asoglu, Mehmet Turker, Abdurrahman Aykut, Seda Jeral Evinc, Ozkan Alan, Mehmet Emin Yilmaz, Ozturk Ates, Hatime Arzu Yasar, Mehmet Kayaalp, Esra Asik, Atila Yildirim, Burcu Bacak, Meltem Baykara, Dicle Yurdatap Koc, Muhammed Bekir Hacioglu, Suleyman Alkan, Ferhat Ekinci, Ahmet Burak Agaoglu, Mesut Yilmaz, Ilhan Hacibekiroglu, Mustafa Karaca, Taliha Guclu Kantar, Gamze Gokoz Dogu, Tuba Karacelik, Melek Karakurt Eryilmaz, Teoman Sakalar, Sedat Biter, Mehmet Mutlu Kıdı, Yasemin Aydınalp Camadan, Mahmut Buyuksimsek

**Affiliations:** 1 Department of Medical Oncology, Faculty of Medicine, Cukurova University, Adana 01330, Turkey; 2 Department of Medical Oncology, Giresun Training and Research Hospital, Giresun 28200, Turkey; 3 Department of Medical Oncology, Adana Training and Research Hospital, Adana 01370, Turkey; 4 Department of Medical Oncology, Cerrahpasa Faculty of Medicine, Istanbul 34098, Turkeyozkan.alan@iuc.edu.tr (O.A.); 5 Department of Medical Oncology, Dr. Abdurrahman Yurtaslan Oncology Training and Research Hospital, Ankara 06200, Turkey; 6 Department of Medical Oncology, Faculty of Medicine, Ankara University, Ankara 06590, Turkey; 7 Department of Medical Oncology, Kanuni Training and Research Hospital, Trabzon 61040, Turkey; 8 Department of Medical Oncology, Faculty of Medicine, Karadeniz Technical University, Trabzon 61080, Turkey; 9 Department of Medical Oncology, Afyon Health Sciences University, Afyonkarahisar 03200, Turkey; burcubacak15@gmail.com (B.B.);; 10 Department of Medical Oncology, Faculty of Medicine, Trakya University, Edirne 22030, Turkeymbekirhacioglu@yahoo.com (M.B.H.); 11 Department of Medical Oncology, Faculty of Medicine, Akdeniz University, Antalya 07070, Turkey; 12 Department of Medical Oncology, Manisa Celal Bayar University, Manisa 45030, Turkeyabagaoglu@hotmail.com (A.B.A.); 13 Department of Medical Oncology, Faculty of Medicine, Sakarya University, Sakarya 54050, Turkey; 14 Department of Medical Oncology, Denizli State Hospital, Denizli 20040, Turkey; 15 Department of Medical Oncology, Pamukkale University, Denizli 20070, Turkey; 16 Department of Medical Oncology, Necmettin Erbakan University, Konya 42090, Turkey; 17 Department of Medical Oncology, Kahramanmaras Sutcu Imam University, Kahramanmaras 46050, Turkey; 18 Department of Medical Oncology, Adana City Hospital, Adana 01370, Turkey

**Keywords:** RAI-R DTC, sorafenib, progression-free survival, radiotherapy, dose reduction, PLR

## Abstract

**Background**: Sorafenib remains an important treatment option for patients with radioiodine-refractory differentiated thyroid cancer (RAI-R DTC). This study evaluated real-world outcomes and prognostic factors in patients treated with sorafenib. **Materials and Methods**: This retrospective multicenter study included 176 patients with RAI-R DTC treated with sorafenib between 2000 and 2024 across sixteen centers. Clinical, pathological and treatment-related variables, including metastatic sites, radiotherapy, dose reduction, inflammatory markers (neutrophil-to-lymphocyte ratio [NLR] and platelet-to-lymphocyte ratio [PLR]) and pretreatment thyroglobulin (Tg), were analyzed. Progression-free survival (PFS) was evaluated using Kaplan–Meier analysis. Prognostic factors were assessed using univariate and multivariate Cox regression analyses. **Results**: The median follow-up duration was 24 months and the median PFS was 21 months (95% CI: 15.5–26.5). Partial response was observed in 82 patients (46.6%), stable disease in 55 (31.3%) and progressive disease in 35 (19.9%). Patients who underwent dose reduction had longer PFS than those without dose reduction (42 vs. 19 months, *p* = 0.030), and absence of dose reduction remained independently associated with progression risk. Patients who received radiotherapy had shorter PFS than those who did not receive radiotherapy (16 vs. 37 months, *p* = 0.002), and radiotherapy-related variables remained independent predictors of progression. Patients with PLR values >138.2 had shorter PFS than those with PLR values ≤ 138.2 (19 vs. 34 months, *p* = 0.047), although this association was not maintained in Cox regression analysis. Similarly, associations between NLR and Tg values and PFS did not reach statistical significance (*p* = 0.112 and *p* = 0.072, respectively). Hand–foot syndrome was the most common toxicity, occurring in 59 patients (33.5%), while Grade 3 hand–foot syndrome was observed in 7 patients (4.0%). **Conclusions**: Sorafenib provided meaningful disease control with a median PFS of 21 months in this real-world cohort. Dose reduction was associated with longer PFS, whereas radiotherapy requirement appeared to reflect a higher-risk subgroup. Toxicities were generally manageable.

## 1. Introduction

Differentiated thyroid carcinoma (DTC), one of the most common endocrine malignancies, accounts for approximately 1–2% of all cancers and represents more than 90% of thyroid malignancies, including papillary, follicular, Hurthle cell and poorly differentiated subtypes [1]. Although most patients with DTC have an excellent prognosis, a subset develops advanced disease with limited treatment options. In this context, radioiodine (RAI) refractoriness—defined as the absence of RAI-avid metastatic lesions or structural disease progression within 6–12 months after the last RAI treatment—represents a critical clinical challenge [2].

Despite these developments, multikinase inhibitors remain the standard first-line treatment for patients with RAI-R DTC [3]. Sorafenib, a multikinase inhibitor targeting tumor proliferation and angiogenesis, has been shown to significantly improve progression-free survival (PFS) compared to placebo [3]. In the DECISION trial, sorafenib achieved a median PFS of 10.8 months compared to 5.8 months in the placebo group [3].

In addition to therapeutic advances, there is increasing interest in identifying prognostic biomarkers that may guide treatment decisions in this heterogeneous patient population [4,5]. Systemic inflammation has been recognized as a key component of cancer progression and several inflammation-based indices have been proposed as prognostic markers [4,5,6]. Among these, the neutrophil-to-lymphocyte ratio (NLR) and platelet-to-lymphocyte ratio (PLR) are among the most widely studied inflammation-based biomarkers in various malignancies [4,5,6]. However, the prognostic value of these markers in patients with RAI-R DTC remains to be clearly defined. In addition to NLR and PLR, other inflammation-related biomarkers such as white blood cell count (WBC), erythrocyte sedimentation rate (ESR) and fibrinogen have also been investigated in thyroid diseases and malignancies [7]. Previous studies have demonstrated significant differences in several inflammatory parameters between benign and malignant thyroid lesions, suggesting a potential relationship between systemic inflammatory response and disease behavior [7]. However, inflammation-based biomarkers are not specific to thyroid cancer and may be influenced by concurrent inflammatory or autoimmune conditions, which should be considered when interpreting their clinical significance [8]. Therefore, the integration of inflammatory markers with clinical and pathological characteristics may provide a more comprehensive assessment of prognosis in patients with thyroid cancer [7,8].

Taken together, these findings highlight the need for better identification of prognostic factors in patients with RAI-R DTC, and this study aimed to evaluate the factors affecting progression-free survival in patients treated with sorafenib, with a particular focus on clinical characteristics and systemic inflammatory markers.

## 2. Materials and Methods

This study was conducted in accordance with the Declaration of Helsinki and was approved by the institutional ethics committee (approval number: 146, date: 26 July 2024).

This retrospective, multicenter study included patients from sixteen centers who were aged ≥ 18 years and diagnosed with progressive, locally advanced, or metastatic RAI-R DTC between 2000 and 2024 and who subsequently received sorafenib treatment. A total of 176 patients with RAI-R DTC who received sorafenib at any stage of their disease course were included. To reflect real-world clinical practice, all eligible patients who received sorafenib were included regardless of treatment line. Eligible patients were required to have measurable or evaluable disease according to RECIST version 1.1, available baseline clinical data and sufficient follow-up information, with disease progression assessed using radiological imaging (CT, MRI and/or PET/CT) [9]. Patients with missing key clinical data, absence of evaluable radiological response, follow-up durations shorter than three months or those receiving concurrent systemic anticancer therapies were excluded from the analysis. RAI-refractory disease was defined as the absence of radioiodine uptake in metastatic lesions, disease progression despite radioiodine uptake or progression within 6–12 months following the last radioactive iodine (RAI) treatment.

Demographic and clinical data were obtained from patient records and institutional databases. Collected variables included age, sex, date of diagnosis, histopathological subtype, tumor localization, metastatic sites, treatment history, radiotherapy (RT) requirement and molecular profiling data when available. Risk stratification was performed according to the American Thyroid Association (ATA) risk stratification system, and patients were classified into low-, intermediate- and high-risk groups [10]. Metastatic sites, including lung, liver, bone and lymph nodes, were recorded at baseline. The number of RAI treatments and cumulative RAI dose were recorded for each patient.

Sorafenib was initiated at a standard dose of 400 mg twice daily. Dose reduction was defined as any decrease in the standard sorafenib dose during treatment due to toxicity or clinical decision. Treatment-related adverse events were evaluated according to the Common Terminology Criteria for Adverse Events (CTCAE), version 5.0 [11].

Tumor response was assessed using RECIST version 1.1 criteria [9]. Progression-free survival (PFS) was defined as the time from initiation of sorafenib treatment to disease progression or death from any cause. Patients without progression were censored at the date of last follow-up. Follow-up duration was calculated from the initiation of sorafenib treatment to the last clinical visit or death.

Neutrophil (10^3^/mL), lymphocyte (10^3^/mL), monocyte (10^3^/mL) and platelet (10^3^/mL) counts were recorded prior to treatment initiation. The neutrophil-to-lymphocyte ratio (NLR) and platelet-to-lymphocyte ratio (PLR) were calculated by dividing neutrophil and platelet counts by lymphocyte count, respectively. The association between inflammatory markers (NLR and PLR) and progression-free survival (PFS) was evaluated. Pretreatment thyroglobulin (Tg) levels were also recorded and evaluated as potential prognostic markers. Cut-off values for Tg, NLR, PLR and PNI were determined using receiver operating characteristic (ROC) curve analysis.

Subgroup analyses were performed based on histopathological subtype, metastatic sites, dose reduction status and inflammatory markers.

Statistical analysis was performed using SPSS 15.0 for Windows (SPSS Inc., Chicago, IL, USA). Descriptive statistics were expressed as numbers and percentages for categorical variables and as means, standard deviations, minimums, maximums and medians for continuous variables. Categorical variables were compared using the Chi-square test. Comparisons of continuous variables between two independent groups were performed using the Mann–Whitney U test because the assumption of a normal distribution was not met. Survival rates were calculated using Kaplan–Meier analysis. Receiver operating characteristic (ROC) curve analysis was performed to determine optimal cutoff values for prognostic markers. Univariate and multivariate Cox regression analyses were performed to identify factors associated with disease progression. Variables with a *p*-value < 0.25 in univariate analysis were included in the multivariate model and backward stepwise selection method was applied. A *p*-value < 0.05 was considered statistically significant.

## 3. Results

A total of 176 patients with RAI-R DTC who received sorafenib were included in the study. The median age was 63 years (range: 52–70), and 52.8% of the patients were female. Papillary carcinoma was the most common histological subtype (71%), followed by follicular carcinoma (22.7%). According to the ATA risk stratification, 96 patients (54.5%) were classified as high risk, while 44 (25.0%) and 36 (20.5%) were categorized as intermediate and low risk, respectively (Table 1).

Sorafenib was administered as first-line therapy in 154 patients (87.5%). The remaining 22 patients (12.5%) had received systemic therapy before sorafenib. Four patients received targeted therapy prior to sorafenib, including Lenvatinib in 1 patient and Vandetanib in 3 patients. Metastasectomy was performed in 44 patients (25%) and 92 patients (52.3%) received RT (Table 2).

Partial response was observed in 82 patients (46.6%), while 55 (31.3%) had stable disease and 35 (19.9%) showed progressive disease. Complete response was observed in 4 patients (2.3%). Dose reduction during treatment was performed in 39 patients (22.2%) (Table 3).

Treatment-related adverse events were observed across multiple categories. Hand–foot syndrome was the most frequently observed toxicity. Grade 3 toxicities were infrequent across all adverse event groups. Treatment discontinuation occurred most commonly due to disease progression, while other causes were rare (Table 4).

**Figure 1:**
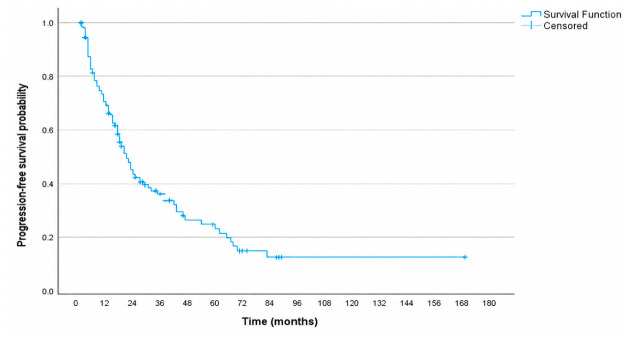
Kaplan–Meier curve for progression-free survival.

The median follow-up duration was 24 months (IQR: 10–45). The median progression-free survival (PFS) was 21 months (95% CI: 15.5–26.5). The estimated PFS rates were 79.4% at 6 months, 67.0% at 1 year, 45.2% at 2 years, 35.8% at 3 years, 22.0% at 5 years and 11.0% at 7 years (Figure 1).

ROC curve analysis identified cut-off values of ≤691 for Tg, >1.5 for NLR, >138.2 for PLR and ≤51.4 for PNI. The corresponding AUC values were 0.523, 0.516, 0.547 and 0.501, respectively. Sensitivity and specificity were 92.4% and 26.2% for Tg, 89.5% and 23.5% for NLR, 68.6% and 47.1% for PLR and 63.1% and 46.7% for PNI. Patients with Tg values ≤ 691 ng/mL had longer PFS compared to those with Tg values > 691 ng/mL (42 months vs. 21 months, *p* = 0.072). In addition, patients with PLR values > 138.2 had shorter PFS compared to those with PLR values ≤ 138.2 (19 months vs. 34 months, *p* = 0.047), while patients with NLR values > 1.5 showed shorter PFS but this difference was not statistically significant (20 months vs. 37 months, *p* = 0.112).

Molecular profiling data were available for a subset of patients. BRAF status was available in 76 patients, which included 42 positive and 34 negative cases. Median PFS was 41 months in patients with positive BRAF status and 22 months in those with negative BRAF status (log-rank *p* = 0.307). NTRK status was available in 57 patients, which included 35 positive and 22 negative cases. Median PFS was 37 months in patients with positive NTRK status and 18 months in those with negative NTRK status (log-rank *p* = 0.505).

Median PFS was 23 months in patients with follicular carcinoma, 22 months in patients with papillary carcinoma and 12 months in patients with undefined histology. Although patients with undefined histology showed numerically shorter PFS, the difference did not reach statistical significance (*p* = 0.692).

No statistically significant association between metastatic sites and PFS was observed (lymph node, *p* = 0.282; lung, *p* = 0.969; liver, *p* = 0.065; bone, *p* = 0.381; other metastatic sites, *p* = 0.189).

**Figure 2:**
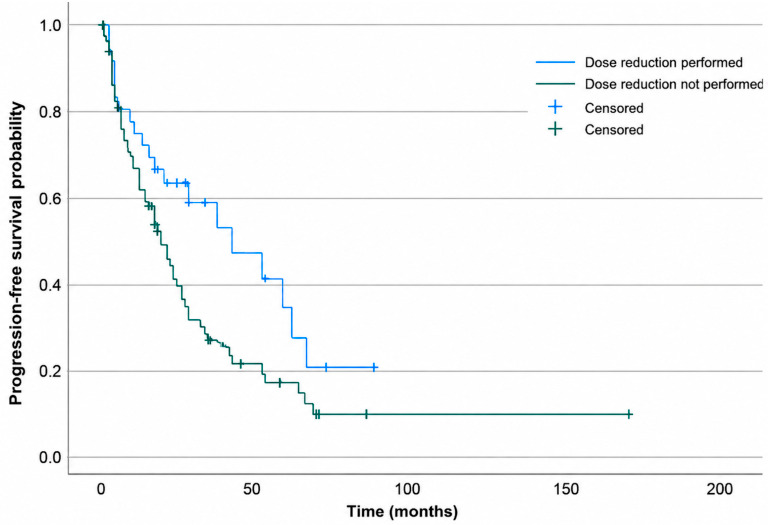
Relationship between dose reduction and progression-free survival.

**Figure 3:**
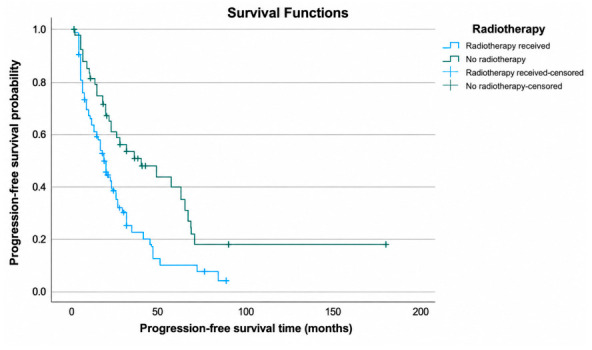
Relationship between radiotherapy status and progression-free survival.

In Kaplan–Meier analysis, patients who underwent sorafenib dose reduction had longer PFS than those without dose reduction (42 months vs. 19 months, *p* = 0.030) (Figure 2). Similarly, patients who did not receive RT had longer PFS compared to those who received RT (37 months vs. 16 months, *p* = 0.002) (Figure 3).

In univariate Cox regression analysis, RT (HR: 1.957, *p* = 0.003), lymph node RT (HR: 2.334, *p* = 0.002), liver RT (HR: 20.537, *p* < 0.001), RT to other metastatic sites (HR: 2.690, *p* = 0.002) and absence of sorafenib dose reduction (HR: 1.730, *p* = 0.034) were associated with increased risk of progression. In multivariate Cox regression analysis using the backward stepwise method, lymph node RT (HR: 3.080, *p* = 0.012), liver RT (HR: 100.77, *p* < 0.001), RT to other metastatic sites (HR: 4.300, *p* = 0.008) and absence of sorafenib dose reduction (HR: 2.563, *p* = 0.022) remained independent predictors of progression (Table 5).

## 4. Discussion

In this multicenter real-world study evaluating patients with RAI-R DTC treated with sorafenib, we demonstrated that sorafenib provided meaningful clinical benefit with a median PFS of 21 months and a high disease control rate. Another notable finding of our study was the significantly longer PFS observed in patients who required dose reduction during treatment, which also remained independently associated with progression risk in multivariate analysis. In addition, patients requiring RT demonstrated less favorable survival outcomes, suggesting that RT requirement may reflect a more advanced disease burden. Furthermore, sorafenib demonstrated a manageable safety profile, with hand–foot syndrome being the most common adverse event and treatment discontinuation occurring predominantly due to disease progression rather than toxicity. On the other hand, the relatively high use of RAI among ATA low-risk patients may reflect the multicenter design of the study, the long study period and variations in RAI administration practices across participating centers.

Lenvatinib is currently considered a preferred first-line treatment option for many patients with RAI-R DTC because of its high response rates and the prolonged progression-free survival demonstrated in clinical trials [12]. Nevertheless, sorafenib remains a relevant therapeutic option in selected patients, particularly when lenvatinib is not available, contraindicated or not well tolerated [13]. Therefore, real-world data regarding sorafenib continue to provide clinically meaningful information for contemporary practice. Our study demonstrated a median PFS of 21 months in patients with RAI-R DTC treated with sorafenib, which appears numerically longer than the 10.8-month median PFS reported in the phase III DECISION trial that led to the approval of sorafenib in this patient population [3]. Similarly, real-world studies such as the RIFTOS MKI study reported a median PFS of 16.7 months in patients receiving sorafenib treatment [14]. In our cohort, the estimated PFS rates remained relatively favorable over time, with 67.0% at 1 year and 45.2% at 2 years, suggesting sustained disease control in a substantial proportion of patients. Although direct cross-study comparisons should be interpreted cautiously due to differences in study design and patient characteristics, our findings support the clinical activity of sorafenib under real-world conditions. The relatively prolonged PFS observed in our cohort may be associated with factors such as multidisciplinary management strategies, close treatment monitoring and the predominantly first-line use of sorafenib in a largely systemic treatment-naive population.

NLR and PLR are widely recognized as biomarkers reflecting the degree of systemic inflammatory response in cancer patients [15,16,17,18]. Increasing evidence has demonstrated that systemic inflammation plays a critical role in tumor progression, angiogenesis and metastatic spread [15,18]. Neutrophils and platelets may contribute to tumor growth through the secretion of proangiogenic and protumoral mediators such as VEGF and TGF-β, whereas lymphocytes represent a key component of the antitumor immune response [19,20,21]. Therefore, elevated NLR and PLR values may reflect a tumor-promoting inflammatory microenvironment accompanied by impaired antitumor immunity. Previous studies in several malignancies, including gastric, ovarian, colorectal and lung cancers, have shown that elevated NLR and PLR are associated with poorer oncological outcomes [15,16,17,18]. In ovarian cancer, pooled analyses demonstrated hazard ratios of 1.63 for overall survival and 1.69 for progression-free survival in patients with elevated NLR. In the present study, elevated PLR values were associated with shorter PFS in patients with RAI-R DTC treated with sorafenib in Kaplan–Meier analysis, although this association did not remain statistically significant in Cox regression analysis. Similarly, elevated NLR values showed a trend toward shorter PFS without reaching statistical significance. These findings suggest that inflammation-based markers may reflect disease biology and tumor-related inflammatory burden, although their independent prognostic value appears limited in this cohort.

Pretreatment Tg levels may also reflect tumor burden and aggressive disease biology in patients with advanced DTC [22]. Previous studies have suggested that elevated Tg levels are associated with advanced-stage disease and poorer oncological outcomes, although their independent prognostic value may become limited in RAI-refractory disease because of tumor dedifferentiation [22,23]. Similarly, in our cohort, patients with higher Tg values demonstrated shorter PFS, although statistical significance was not reached.

Patients who undergo dose reduction tend to remain on treatment for longer periods and consequently have a higher likelihood of developing cumulative toxicities requiring dose modification over time [24]. Real-world studies have similarly demonstrated that dose modification is common during tyrosine kinase inhibitor treatment [23]. In the RIFTOS MKI study, approximately 70% of patients receiving sorafenib required dose modification and maintained a median PFS of 16.7 months [14]. Consistent with these observations, patients who underwent dose reduction in our cohort demonstrated significantly longer PFS compared to those without dose modification in Kaplan–Meier analysis (42 months vs. 19 months, *p* = 0.030). Moreover, absence of dose reduction remained independently associated with progression risk in multivariate Cox regression analysis.

Sorafenib is generally considered to have a manageable safety profile in the treatment of RAI-R DTC [25]. Previous clinical studies have identified hand–foot syndrome (palmar–plantar erythrodysesthesia) as the most common adverse event, which occurred in approximately 76% of patients [24]. However, the rates of severe Grade 3–4 hand–foot syndrome (20%) and other Grade 3 toxicities such as diarrhea (6%) and fatigue (6%) have been reported to be relatively low, supporting the tolerability of the treatment [25]. Real-world studies and phase III trials have further demonstrated that approximately 64–70% of patients require dose modification because of adverse events; nevertheless, disease progression rather than toxicity remains the leading cause of permanent treatment discontinuation [14,25,26]. Consistent with the literature, hand–foot syndrome was also the most frequently observed toxicity in our cohort, while Grade 3 toxicities were relatively uncommon across adverse event groups. In addition, treatment discontinuation occurred predominantly because of disease progression. Overall, our findings support the view that sorafenib has a predictable and manageable toxicity profile that may allow for prolonged treatment continuation with appropriate supportive care and dose management strategies.

The prognostic impact of histopathological subtypes and metastatic sites in patients with RAI-refractory DTC treated with sorafenib remains controversial. In our cohort, no statistically significant differences in PFS were observed between histopathological subtypes (*p* = 0.692). Similarly, metastatic sites were not significantly associated with PFS, although patients with liver metastasis demonstrated numerically shorter PFS compared to those without liver involvement (16 months vs. 23 months, *p* = 0.065). Previous studies evaluating sorafenib in patients with RAI-refractory DTC have likewise reported no clinically significant differences in progression-free survival between histopathological subtypes such as papillary, follicular, Hurthle cell or poorly differentiated tumors [27]. These findings suggest that sorafenib may retain therapeutic activity across different histological subtypes of RAI-refractory disease [27].

Regarding metastatic sites, the presence of distant metastases, particularly those detected by [18F] FDG PET/CT, has been reported as an adverse prognostic factor for PFS [22]. Although some studies have suggested a borderline negative impact of liver metastasis on survival outcomes, these findings do not appear to outweigh the overall systemic activity of sorafenib [14]. In addition, RT, particularly external beam radiotherapy (EBRT), is generally used in patients with symptomatic lesions, unresectable disease or high-risk metastatic involvement requiring local control [28]. Similarly, in our cohort, patients requiring radiotherapy demonstrated significantly shorter PFS and radiotherapy-related variables remained independently associated with progression risk in multivariate analysis. These findings likely reflect a subgroup with greater disease burden and more aggressive tumor biology rather than a direct adverse effect of radiotherapy itself. Furthermore, the very high hazard ratio observed for liver irradiation should be interpreted cautiously, as the limited number of patients in this subgroup may have influenced the stability of the model. Overall, our findings support the concept that sorafenib may provide disease control across different metastatic subgroups of RAI-refractory DTC.

## 5. Limitations

This study has several limitations. First, its retrospective multicenter design may have introduced selection bias and interinstitutional heterogeneity in patient management and follow-up strategies. Due to the multicenter design, the complete patient flow from radioiodine treatment to radioiodine-refractory disease and subsequent sorafenib treatment was not available and the interval between surgery and first RAI administration could not be consistently evaluated across participating centers. Another limitation of this study is the lack of comprehensive overall survival data across participating centers, which precluded a reliable analysis of overall survival outcomes.

A further limitation is that the association between dose reduction and prolonged PFS should be interpreted with caution, as dose modifications are more likely to occur in patients who remain progression-free and continue treatment for longer periods. In addition, radiotherapy indications and supportive treatment approaches were not fully standardized across centers because treatment decisions were based on real-world multidisciplinary clinical practice. The wide confidence intervals observed for some variables in the multivariate analysis, particularly liver irradiation, also suggest that these findings should be interpreted with caution. Finally, radiological response assessments were performed within routine clinical practice, which may have introduced variability in response evaluation.

## 6. Conclusions

In conclusion, sorafenib demonstrated meaningful clinical activity and a manageable safety profile in patients with RAI-refractory differentiated thyroid cancer in this multicenter real-world cohort. Patients who underwent dose reduction showed longer progression-free survival, suggesting that appropriately managed treatment modification may support prolonged treatment benefit. In addition, radiotherapy requirement was associated with poorer survival outcomes and remained independently associated with progression risk, likely reflecting greater disease burden and more aggressive tumor biology. Overall, our findings support the continued role of sorafenib as an effective treatment option in patients with RAI-refractory DTC.

## Figures and Tables

**Table 1:** Baseline and Disease Characteristics of Patients (*n* = 176).

Variable	Value *
Demographics	
Age, years (median, IQR)	63 (52–70)
Female	93 (52.8)
Male	83 (47.2)
Histopathology	
Papillary carcinoma	125 (71.0)
Follicular carcinoma	40 (22.7)
Undefined	11 (6.3)
ATA Risk Stratification	
Low risk	36 (20.5)
Intermediate risk	44 (25.0)
High risk	96 (54.5)
Surgical Treatment	
Lobectomy	17 (9.7)
Total thyroidectomy	88 (50.0)
Total thyroidectomy + BD	61 (34.7)
Other	10 (5.7)
Metastatic Sites (at baseline) **	
Lymph node	87 (49.4)
Lung	113 (64.2)
Liver	17 (9.7)
Bone	72 (40.9)
Other	14 (8.0)
RAI Treatment	
Number of RAI courses (median, IQR)	5 (4–5.3)
Cumulative RAI dose, mCi (median, IQR)	600 (375–900)

Abbreviations: ATA, American Thyroid Association; RAI, radioactive iodine; BD, bilateral dissection; mCi, millicurie. * Categorical variables are presented as *n* (%) and continuous variables are presented as median (IQR: Interquartile Range). ** Some patients have more than one metastatic site.

**Table 2:** Treatment Characteristics of Patients.

Variable	Value, *n* (%)
Line of Sorafenib Therapy	
First-line	154 (87.5)
Second-line	19 (10.8)
Third-line	2 (1.1)
Fourth-line	1 (0.6)
Pre-sorafenib Systemic Therapy	
None	154 (87.5)
Carboplatin–taxane	7 (4.0)
Anthracycline	8 (4.5)
Other chemotherapy	3 (1.7)
Targeted therapy before sorafenib	4 (2.3)
Local Treatments	
Metastasectomy performed	44 (25)
Metastasectomy not performed	132 (75)
Radiotherapy	
Radiotherapy received	92 (52.3)
Radiotherapy not received	84 (47.7)
Sites of Metastasectomy	
Lymph node	18 (10.2)
Lung	6 (3.4)
Liver	3 (1.7)
Bone	13 (7.4)
Other	4 (2.3)
Not applicable	132 (75.0)
Sites of Radiotherapy	
Lymph node	22 (12.5)
Lung	9 (5.1)
Liver	2 (1.1)
Bone	59 (33.5)
Other	13 (7.4)
Not applicable	71 (40.3)

**Table 3:** Response to Sorafenib and Dose Modification.

Variable	Value, *n* (%)
Best Response to Sorafenib	
Complete response	4 (2.3)
Partial response	82 (46.6)
Stable disease	55 (31.3)
Progressive disease	35 (19.9)
Dose Reduction	
Performed	39 (22.2)
Not performed	137 (77.8)

**Table 4:** Sorafenib-Related Toxicity Profile.

Variable	Value, *n* (%)
Diarrhea	
No toxicity	139 (79.0)
Grade 1	25 (14.2)
Grade 2	6 (3.4)
Grade 3	6 (3.4)
Hepatitis	
No toxicity	164 (93.2)
Grade 1	8 (4.5)
Grade 2	3 (1.7)
Grade 3	1 (0.6)
Pneumonitis	
No toxicity	173 (98.3)
Grade 1	1 (0.6)
Grade 2	1 (0.6)
Grade 3	1 (0.6)
Stomatitis	
No toxicity	150 (85.2)
Grade 1	17 (9.7)
Grade 2	9 (5.1)
Skin Toxicity	
No toxicity	118 (67.0)
Grade 1	31 (17.6)
Grade 2	22 (12.5)
Grade 3	5 (2.8)
Hand–Foot Syndrome	
No toxicity	117 (66.5)
Grade 1	26 (14.8)
Grade 2	26 (14.8)
Grade 3	7 (4.0)
Renal Function Impairment	
No toxicity	153 (86.9)
Grade 1	19 (10.8)
Grade 2	2 (1.1)
Grade 3	2 (1.1)
Treatment Status and Reasons for Discontinuation	
Ongoing	16 (9.1)
Due to progression	156 (88.6)
Congestive cardiac failure	1 (0.6)
Treatment refusal	1 (0.6)
Osteonecrosis of jaw	1 (0.6)
Acute myeloid leukemia	1 (0.6)

**Table 5:** Univariate and multivariate Cox regression analyses for progression risk.

Variable	Univariate *p*	Univariate HR	95% CI	Multivariate *p*	Multivariate HR	95% CI	Included in Multivariate
Age	0.125	1.013	0.996–1.030	0.051	1.035	1.000–1.071	Yes
Papillary histology	0.692	1.101	0.685–1.767	-	-	-	No
Undefined histology	0.399	1.418	0.630–3.193	-	-	-	No
Low ATA risk	0.848	0.933	0.460–1.891	-	-	-	No
Intermediate ATA risk	0.835	0.951	0.590–1.532	-	-	-	No
Liver metastasis	0.072	1.661	0.956–2.886	-	-	-	Yes
Radiotherapy	0.003	1.957	1.249–3.064	-	-	-	Yes
Lymph node radiotherapy	0.002	2.334	1.376–3.957	0.012	3.080	1.277–7.431	Yes
Liver radiotherapy	<0.001	20.537	4.145–101.756	<0.001	100.77	8.181–1241.1	Yes
Other site radiotherapy	0.002	2.690	1.435–5.040	0.008	4.300	1.463–12.639	Yes
No dose reduction	0.034	1.730	1.043–2.872	0.022	2.563	1.146–5.730	Yes
Tg ≤ 691 ng/mL	0.083	2.093	0.909–4.819	0.065	3.220	0.928–11.165	Yes
NLR > 2.7	0.120	1.647	0.879–3.086	-	-	-	Yes
PLR > 138.2	0.051	1.523	0.998–2.323	-	-	-	Yes

Abbreviations: HR, hazard ratio; CI, confidence interval; ATA, American Thyroid Association; Tg, thyroglobulin; NLR, neutrophil-to-lymphocyte ratio; PLR, platelet-to-lymphocyte ratio.

## Data Availability

The data presented in this study are available on reasonable request from the corresponding author.
